# Comprehensive Mutation Analysis for Congenital Muscular Dystrophy: A Clinical PCR-Based Enrichment and Next-Generation Sequencing Panel

**DOI:** 10.1371/journal.pone.0053083

**Published:** 2013-01-11

**Authors:** C. Alexander Valencia, Arunkanth Ankala, Devin Rhodenizer, Shruti Bhide, Martin Robert Littlejohn, Lisa Mari Keong, Anne Rutkowski, Susan Sparks, Carsten Bonnemann, Madhuri Hegde

**Affiliations:** 1 Department of Human Genetics, Emory University School of Medicine, Atlanta, Georgia, United States of America; 2 Division of Human Genetics, Cincinnati Children's Hospital Medical Center, Cincinnati, Ohio, United States of America; 3 Department of Pediatrics, University of Cincinnati Medical School, Cincinnati, Ohio, United States of America; 4 Cure CMD and Kaiser SCPMG, Los Angeles, California, United States of America; 5 Carolinas Medical Center, Charlotte, North Carolina, United States of America; 6 Neuromuscular and Neurogenetic Disorders of Childhood Section, Neurogenetics Branch, National Institute of Neurological Disorders and Stroke, National Institutes of Health, Bethesda, Maryland, United States of America; Tel Aviv University, Israel

## Abstract

The congenital muscular dystrophies (CMDs) comprise a heterogeneous group of heritable muscle disorders with often difficult to interpret muscle pathology, making them challenging to diagnose. Serial Sanger sequencing of suspected CMD genes, while the current molecular diagnostic method of choice, can be slow and expensive. A comprehensive panel test for simultaneous screening of mutations in all known CMD-associated genes would be a more effective diagnostic strategy. Thus, the CMDs are a model disorder group for development and validation of next-generation sequencing (NGS) strategies for diagnostic and clinical care applications. Using a highly multiplexed PCR-based target enrichment method (RainDance) in conjunction with NGS, we performed mutation detection in all CMD genes of 26 samples and compared the results with Sanger sequencing. The RainDance NGS panel showed great consistency in coverage depth, on-target efficiency, versatility of mutation detection, and genotype concordance with Sanger sequencing, demonstrating the test's appropriateness for clinical use. Compared to single tests, a higher diagnostic yield was observed by panel implementation. The panel's limitation is the amplification failure of select gene-specific exons which require Sanger sequencing for test completion. Successful validation and application of the CMD NGS panel to improve the diagnostic yield in a clinical laboratory was shown.

## Introduction

The main objective of human genetics is to identify the genetic variants underlying specific phenotypes and provide molecular diagnoses to guide clinical management [1]. Genetic heterogeneity in inherited disorders including breast cancer, intellectual disability, ataxia, hearing loss, immunodeficiency, cardiomyopathies, and inherited muscle disorders such as the congenital muscular dystrophies has driven the development of novel screening and testing approaches [2]. Over the past decade, our molecular understanding of the congenital muscular dystrophies (CMDs) has expanded greatly [3]. CMDs are rare genetic muscle disorders that present early at birth or within the first 2 years of life, with variable inheritance patterns. Generally, they are characterized by congenital hypotonia, delayed motor development, progressive muscle weakness, and dystrophic features on muscle biopsy [4]. CMDs are genetically and phenotypically heterogeneous and include disorders caused by: 1) recessive or dominant *COL6A1*, *COL6A2*, and *COL6A3* mutations, which manifest as Ullrich or Bethlem CMD; 2) recessive *LAMA2* mutations, which result in merosin-deficient CMD (MDC1A); 3) recessive mutations in the *POMT1*, *POMT2*, *POMGNT1*, *FKRP*, *FKTN*, or *LARGE* genes, which manifest as purely muscular or syndromic conditions, such as Fukuyama CMD, muscle-eye-brain disease (MEB), Walker-Warburg syndrome (WWS), or CMD type 1C (MDC1C); 4) recessive mutations in the *SEPN1* gene, which manifest as rigid-spine syndrome (RSS); 5) dominant lamin A/C mutations, which result in the congenital form of Emery Dreifuss muscular dystrophy; and 6) recessive *ITGA7* mutations, which manifest as CMD with integrin A7 deficiency [4], [5]. Novel genes remain to be identified in patients with clinical collagen VI-like, dystroglycanopathy-like and not previously described phenotypes with congenital onset and dystrophic muscle biopsy findings. In a recent survey of a national UK referral service for CMD diagnostics, a genetic diagnosis was reached in 53 of 116 patients, with most common diagnoses being collagen VI related disorders (19%), dystroglycanopathy (12%) and merosin deficient congenital muscular dystrophy (10%) [6].

Diagnosing a specific CMD subtypes may present a challenge and requires a multidisciplinary expertise (neurology, pathology, genetics, and neuroradiology). Currently, there are only a few centers with the expertise to recognize and delineate the wide range of overlapping clinical features [4], [7], [8]. Even then, patients undergo a battery of immunostains and individual gene sequencing analyses to arrive at an exact diagnosis [9]. The most frequently used CMD diagnostic method is sequential gene-by-gene mutation detection by Sanger sequencing. Sequencing a gene is rightly the preferred approach for mutation detection in CMD, since most mutations identified to date are either point mutations or small insertions and deletions. However, the difficulty in disease delineation and the increasing number of genes implicated mandate a more comprehensive molecular diagnostic approach to improve both diagnostic and cost efficiencies.

Within the last 5 years, high-throughput sequencing technology, referred to as next-generation sequencing (NGS) has successfully identified mutations in novel genes for a number of conditions, including Sensenbrenner syndrome, Kabuki syndrome, and Miller syndrome [10]–[12]. NGS facilitates target re-sequencing for rapid, accurate and lower cost diagnostic applications. However, with several target enrichment strategies, including microarray-based capture, in-solution capture, and polymerase chain reaction (PCR)-based amplification [13]–[20], and NGS sequencing platforms, such as Roche 454 GS FLX, Illumina Genome Analyzer, Applied Biosystems SOLiD, Helicos Biosciences HeliScope, and Pacific Biosciences SMRT, being commercially available, selection and validation of the technologies becomes crucial [21], [22].

In this study, we describe the development and validation of an NGS panel, using RainDance (as enrichment technology) and Applied Biosystems SOLiD3 (as sequencing platform), for comprehensive mutation detection in CMD genes, along with its diagnostic yield and clinical implementation in patients with confirmed diagnosis, serving as positive controls, and with clinically suspected CMD (Table 1).

**Table 1:** Summary of the clinical features of CMD patients.

Sample	Age	Sex	Ethnicity	Clinical presentation
CMD-1	-	-	-	CMD XX-XX
CMD-2	-	-	-	CMD XX-XX
CMD-3	-	-	-	CMD XX-XX
CMD-4	-	-	-	CMD XX-XX
CMD-5	-	-	-	CMD XX-XX
CMD-6	-	-	-	CMD XX-XX
CMD-7	-	-	-	CMD XX-XX
CMD-8	8	M	ND	High CK
CMD-9	30	M	East Indian	Myogenic by IHC, high CK
CMD-10	6	M	Hispanic	Gower's sign, DD, muscle weakness, normal MRI and CK
CMD-11	1	F	African American	High CK
CMD-12	12	M	Caucasian	Muscle weakness, high CK
CMD-13	-	-	-	CMD XX-XX
CMD-14	-	-	-	CMD XX-XX
CMD-15	-	-	-	CMD XX-XX
CMD-16	-	-	-	CMD XX-XX
CMD-17	-	-	-	CMD XX-XX
CMD-18	-	-	-	Ophthalmoplegia
CMD-19	-	-	-	CMD XX-XX
CMD-20	-	-	-	CMD XX-XX
CMD-21	-	-	-	CMD XX-XX
CMD-22	-	-	-	High CK, normal MRI
CMD-23	-	-	-	High CK, on mechanical ventilators
CMD-24	-	-	-	CMD XX-XX
CMD-25	-	-	-	CMD XX-XX
CMD-26	-	-	-	High CK, WM abnormality on MRI, facial weakness

NP, not performed; -, no data available; CSF, cerebrospinal fluid; IHC, immunohistochemistry; CK, creatinine kinase; DD, developmental delay; CMD XX-XX, possible diagnosis of CMD.

## Results

### Sequencing yields and optimal target base coverage

The targeted next-generation sequencing panel was designed to amplify all exons of the 12 known CMD associated genes (Table 2). The overall target enrichment and next-generation sequencing yielded an average of 1,195,183 reads; 57% of these reads mapped to the genome and 35% mapped to the targeted regions. The total coverage of all targeted bases ranged from 87 to 94% at 5× and from 84 to 94% at 20×. The mean gene depth of coverage across all samples ranged from 0× for *POMT1* to 108× for *POMGNT1*, with an average of 50×. Despite the high mean gene read depth and target region coverage, several exons, including exon 1 of *SEPN1* and all exons of *POMT1* had no mapped reads [23].

**Table 2:** CMD-associated genes included in the clinical CMD NGS panel.

Gene	Associated clinical syndrome	No. reported mutations (HGMD)	Transcript size	No. coding exons	No. amplicons for Sanger sequencing
*LAMA2*	Merosin-deficient congenital muscular dystrophy (CMD1A)	127	9708	65	65
*FKRP*	Fukutin-related proteinopathy (MDC1C), muscle-eye-brain disease (MEB), Walker-Warburg syndrome (WWS), LGMD2I, FCMD	79	1488	1	1
*LARGE*	LARGE-related congenital muscular dystrophy (MDC1D)	9	2268	16	16
*FKTN*	Fukuyama congenital muscular dystrophy (FCMD), Walker-Warburg syndrome (WWS)	39	1383	11	11
*POMT1*	Walker-Warburg syndrome (WWS), LGMD2K	55	2241	20	20
*POMT2*	Muscle-eye-brain disease (MEB), Walker-Warburg syndrome (WWS)	35	2250	21	21
*POMGNT1*	Muscle-eye-brain disease (MEB)	50	1980	22	22
*SEPN1*	Rigid spine muscular dystrophy	43	1770	13	13
*COL6A1*	Ulrich congenital muscular dystrophy and Bethlem myopathy	38	3084	35	35
*COL6A2*	Ulrich congenital muscular dystrophy and Bethlem myopathy	66	3057	28	28
*COL6A3*	Ulrich congenital muscular dystrophy and Bethlem myopathy	31	9531	44	44
*ITGA7*	Merosin-positive congenital muscular dystrophy	4	3411	25	25

Of all the exons targeted, 49 consistently showed less than 20× average coverage across all samples, which could be due largely to sequence complexity, problematic library synthesis, or unusual GC content of the fragments (Table 3). *SEPN1*, *COL6A1*, and *POMT2* had very high GC content, namely, 87%, 73%, and 73%, respectively, specifically in their first coding exon, while *POMT1* had an average of 56% GC content across all exons. In contrast, exons 34, 13, 6, 7, and 27, of *COL6A1*, *COL6A3*, *FKTN*, *FKTN*, and *LAMA2*, respectively, had very low GC content and consequently had no mapped reads in the NGS data.

**Table 3:** Exons with consistently low coverage (<20× average) across all samples.

Gene	Exon number
*COL6A1*	1, 5, 12, 24, 30, 34, 35
*COL6A2*	3, 6, 7, 14, 16, 22, 24, 26, 27
*COL6A3*	13, 15
*FKTN*	6, 7
*ITGA7*	15, 25
*LAMA2*	1, 27, 44, 47
*POMT1*	2, 3, 4, 5, 6, 7, 8, 9, 10, 11, 12, 13, 14, 15, 16, 17, 18, 19, 20
*POMT2*	9, 10
*SEPN1*	1, 6

### High variant detection rates in targeted regions with excellent coverage, phred scores, and allele percentages

Known variants/mutations in the 5 variant-positive control samples re-detected by the CMD NGS test had coverage, Phred score, and allele percentages well above acceptable thresholds (Table 4). This included all previous Sanger-detected variants in exons not problematic for NGS. As expected, none of these variants were detected in the wild-type control (CMD-13) sample. However, additional variants (ranging from 0–14 per sample), not confirmed by Sanger sequencing, passed these threshold values and are thus false positives (Table 5). These false-positive calls are due to several factors including low coverage, low Phred scores and skewed allele percentages of those specific genomic areas. As shown in the Table 5, the false-positive rates ranged from 0% in CMD-11 to 37% in CMD-8. Overall, a total of 85 variants were detected in all 5 variant-positive control samples, of which 68 are reported in the dbSNP database and are, thus, true variants. This in turn reflects the low false-positive rate of the targeted approach via this panel test. Similarly, in the 20 blinded samples, 271 variants were detected, of which 98 are dbSNP calls (Table 6).

**Table 4:** Validation of NGS for variant/mutation detection on known CMD-positives samples.

Sample	Gene	Mutations/variants detected by Sanger sequencing	Mutation/variant type	Detected by NGS	Coverage	Mutant allele %
CMD-8	*COL6A1*	c.1931G>A (p.R644Q), Het	missense	−	11	-
	*COL6A2*	c.1770G>C (p.T590), Het	silent	+	8	25
	*COL6A2*	c.2994C>T (p.H998), Het	silent	+	20	50
CMD-9	*COL6A1*	IVS29-8G>A, Het	intronic	+	7	71
	*FKTN*	IVS9-40C>A, Het	intronic	(−)	-	-
	*LAMA2*	c.2084C>T (p.D695V), Het	missense	+	82	52
	*LAMA2*	c.5614G>T (p.D1872Y), Het	missense	+	29	31
	*SEPN1*	IVS5+39C>T, Het	intronic	(−)	-	-
	*SEPN1*	IVS11-31C>T, Het	intronic	(−)	-	-
	*SEPN1*	c.1645G>A (p.V549M), Het	missense	+	78	38
	*SEPN1*	c.1773+44G>T, Het	3′ UTR	−	-	-
CMD-10	*COL6A1*	IVS26+50C>T, Het	intronic	(−)	-	-
	*COL6A1*	c.2424G>T (p.Q808H), Het	missense	−	17	-
	*COL6A2*	IVS24-3dupC, Het	duplication	−	8	-
	*COL6A3*	IVS38-34C>T, Homo	intronic	(−)	-	-
CMD-11	*LAMA2*	c.3154A>G, Het	splicing	+	17	58
	*LAMA2*	c.6617delT, Het	deletion	−	1	-
CMD-12	*POMGNT1*	c.636C>T (p.F212), Het	splicing^a^	+	34	29
	*POMGNT1*	IVS17+1G>A, Het	splicing^a^	+	105	55
CMD-13	-	-	-	−	-	-

a This mutation has been reported in individuals with MEB disease; +, detected; −, Not detected; (−) Mutation and/or variant not detected because the bioinformatics algorithm for NGS data is set to detect +/−20 bases from exon/intron boundaries.

**Table 5:** Sequence variants identified by NGS sequencing in control samples.

Sample	Sanger-established variants	Total NGS variants	dbSNP variants	Non - dbSNP variants	Filtered variants	False positive rate^a^ (%)
CMD-8	5	8	6	2	15	37
CMD-9	39	53	44	9	24	26
CMD-10	12	14	12	2	19	14
CMD-11	4	4	4	0	9	0
CMD-12	4	6	2	4	0	33

a Represents percentage of variants that were detected by NGS but not confirmed by Sanger sequencing; Total NGS Variants, Include variants that passed threshold settings described in text; Non-dbSNP Variants, NGS variants not listed in dbSNP; Filtered Variants, Variants that were initially detected by NGS but did not meet thresholds and got filtered by the criteria described in Figure 2.

**Table 6:** Sequence variants identified by RainDance enrichment and NGS.

Sample	Total NGS variants	dbSNP variants	Non-dbSNP variants	Filtered variants	Sanger-confirmed variants
CMD-1	18	8	10	10	8
CMD-2	15	12	3	6	8
CMD-3	19	10	9	7	11
CMD-4	17	8	9	9	11
CMD-5	24	11	13	6	14
CMD-6	15	8	7	18	8
CMD-7	20	13	7	8	12
CMD-14	4	1	3	2	2
CMD-15	10	3	7	1	5
CMD-16	17	5	12	7	6
CMD-17	10	7	3	9	5
CMD-18	11	7	5	2	8
CMD-19	10	3	7	1	5
CMD-20	16	0	16	0	4
CMD-21	14	0	14	2	2
CMD-22	7	0	7	1	1
CMD-23	18	2	16	1	3
CMD-24	11	0	11	0	3
CMD-25	9	0	9	1	4
CMD-26	6	0	6	0	1
Total	271	98	174	91	121

Total NGS Variants, Include variants that passed threshold settings described in text; Non-dbSNP Variants, NGS variants not listed in dbSNP; Filtered Variants, Variants that were initially detected by NGS but did not meet thresholds and were filtered by the criteria described in Figure 2.

### Versatility of variant detection

**Figure 1:**
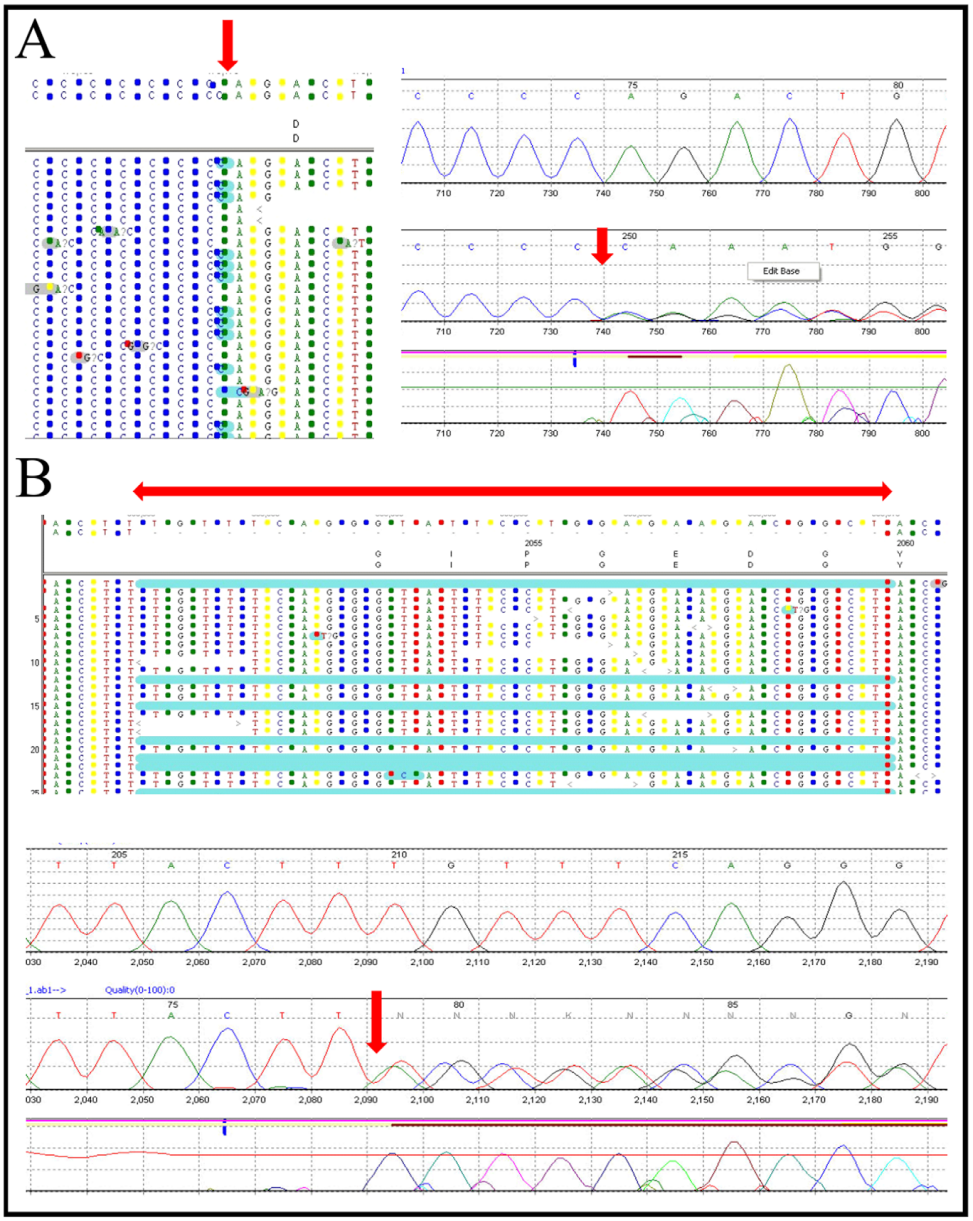
Variant detection by NGS (top) and Sanger sequencing confirmation (bottom). (*A*) Example of an insertion mutation: IVS15-3-2insC in *COL6A2* identified byNGS (to the left) and confirmed by Sanger sequencing (to the right). (*B*) Example of a 30-bp deletion: IVS16-8del30 in *COL6A3* as represented by NGS analysis (top panel) and Sanger sequencing (bottom panel). The exact site or representation of the mutation is indicated by the red arrowheads.

The ability of NGS to efficiently detect all kinds of mutations, including point mutations and small insertions/deletions, was interrogated using previously Sanger-confirmed variants or mutations in the variant-positive samples (Table 4). These variant-positive samples represented all the different types of variants NGS is expected to detect. These included silent, c.1770G>C (p.T590) (CMD-8); missense, c.2084C>T (p.D695V) (CMD-9); small deletion, c.6617delT (CMD-11); and small duplication, IVS24-3dupC (CMD-10) variants in genes *COL6A2*, *LAMA2*, *COL6A3*, and *COL6A2*, respectively (Table 4). Some of these insertion and deletion variants, as detected by NGS and confirmed by conventional Sanger sequencing, are represented in Figure 1. Additionally, potential causative mutations and variants of different types were identified in blinded samples and were concordant with previous Sanger sequencing results obtained in Dr. Carsten Bonnemann's laboratory (Table 7). All confirmed variants had a coverage of at least 7× and mutant allele percentage of 17–71% for heterozygous and 78–100% for homozygous variants (Tables 4 and 7).

**Table 7:** Potential causative variants and mutations identified in the blinded clinical samples.

Sample	Gene	Mutations/variants detected by NGS (blinded)	NGS coverage	Mutant allele % by NGS	Associated CMD sub-type
CMD-1	*COL6A3*	IVS14-8_-29_delTGTTTCAGGGTATTCCTGGAGAAGACGGC, (het)	87	16	UCMD
CMD-2		None Detected	-	-	-
CMD-3	*COL6A2*	c.1402C>T; p.R468X, (het)	108	51	UCMD
CMD-4	*COL6A3*	c.53C>A; p.A18X, (homo)	111	94	UCMD
CMD-5		None Detected	-	-	-
CMD-6	*COL6A1*	IVS21-2A>G, (het)			UCMD
CMD-7	*COL6A1*	IVS14+1G>A, (het)	19	68	UCMD
CMD-14	*COL6A2*	Whole Gene Deletion	-	-	UCMD
CMD-15		None Detected	-	-	-
CMD-16		None Detected	-	-	-
CMD-17		None Detected	-	-	-
CMD-18		None Detected	-	-	-
CMD-19	*COL6A2*	IVS24-9G>A, (het)	14	21	UCMD
CMD-20	*LAMA2* *LAMA2*	c.652_653_delTT (het);c.2230C>T, p.R744X (het)	6894	4440	MDC1A
CMD-21		None Detected	-	-	-
CMD-22		None Detected	-	-	-
CMD-23		None Detected	-	-	-
CMD-24	*LAMA2*	c.1580G>A; p.C527Y, (homo)	487	94	MDC1A
CMD-25	*LAMA2* *LAMA2*	c.4048C>T; p.R1350X, (het)c.1580G>A; p.C527Y, (het)	96319	4550	MDC1A
CMD-26		None Detected	-	-	-

### Multiple parameters as data filters for identification of causative mutations

**Figure 2:**
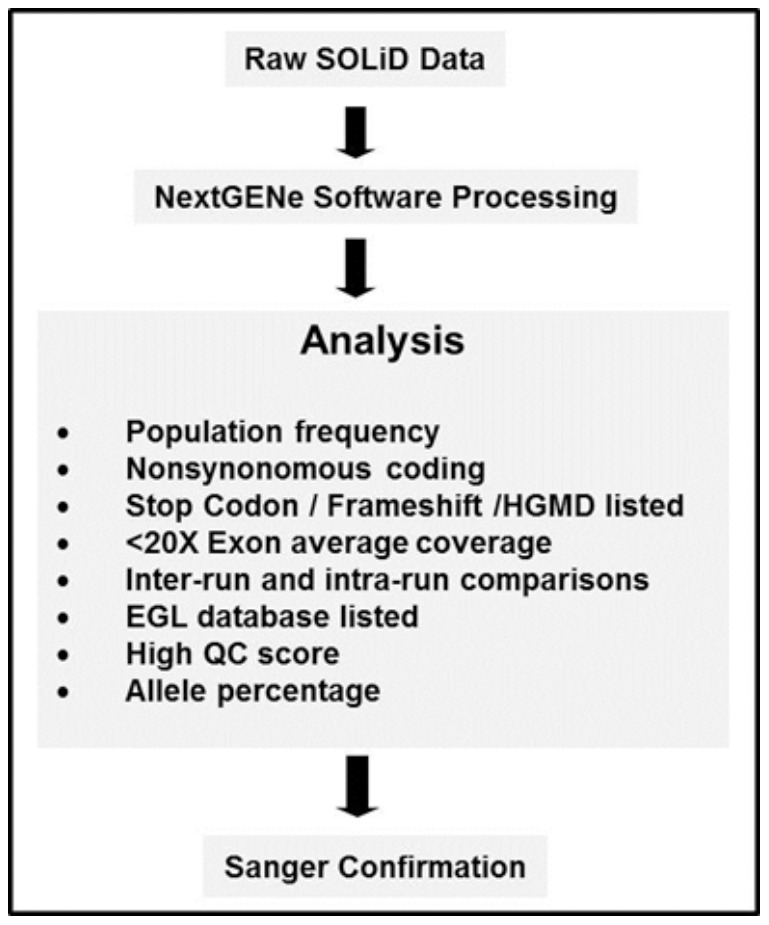
Schematic diagram of the analysis workflow. The flowchart demonstrates the criteria used to select variants that were Sanger sequencing confirmed. In essence, selected variants that had >20× coverage, a low allele frequency, and nonsynonymous changes were Sanger confirmed if they were listed on HGMD, frameshift, or nonsense changes. In addition, interesting variants with a coverage <20× were also confirmed (Table 7).

For clinical applications, it is crucial to reduce the number of variants that need confirmation by Sanger sequencing (lower false-positive rate) to maintain an acceptable cost-benefit ratio. This was evaluated with the variant call data from known positive samples (CMD-8 to CMD-12) by including multiple parameters for variant filtering as illustrated in Figure 2. Following this, variants with coverage less than 20× were all filtered out, unless they were listed in HGMD, dbSNP, or EGL (Emory Genetics Laboratory) databases as definitive known pathogenic variants or mutations. Similarly, variants with high frequency (observed in multiple samples) were removed, unless they were found to be in HGMD, frameshift, or nonsense changes. Additionally, synonymous variants, dbSNP variants with allele frequency >1%, were filtered out of the list as well. When the coverage is greater than 20×, variant calls with mutant allele percentages greater than 85% and less than 30% were retained as homozygous and heterozygous calls, respectively, and others were filtered out. Simultaneous consideration of the multiple parameters described above, for data filtering, significantly reduced the number of variant calls requiring Sanger confirmation (Table 5). Using these parameters, all potential disease-causing mutations previously identified by Carsten Bonnemann's laboratory, but initially blinded to EGL staff, were detected in the 20 blinded clinical samples (Table 7).

### NGS panel approach has higher clinical yield compared to a sequential Sanger sequencing approach

Since the NGS CMD panel was made available at EGL, a number of tests have been ordered and yielded a higher call percentage than single-gene tests (Table 8). Call percentage refers to the identification and report of pathogenic mutations in the respective gene. For example, *LAMA2* and *FKTN* yielded the highest percentages for the single-gene test, 64% and 35%, respectively. In contrast, panels 1 and 2, containing *LAMA2* and *FKTN*, had call percentages of 54% and 94%. Generally, the total percentage call for the gene-by-gene approach was 17% compared to the higher total call percentage of 41% obtained by the 4 NGS panels. The low call percentage or mutation detection rate for individual gene tests is due mostly to incorrect presumption of genetic etiology and suspicion of the incorrect gene.

**Table 8:** Comparison of clinical yields of single-gene versus NGS panel tests.

	Test code	Gene	No. of patients tested	No. of pathogenic calls made	Call %^a^
**Individual gene test by Sanger sequencing**					
1	SC6A1	*COL6A1*	14	0	0
2	SC6A2	*COL6A2*	14	1	7
3	SC6A3	*COL6A3*	13	0	0
4	SFKRP	*FKRP*	25	3	12
5	SFKTN	*FKTN*	26	9	35
6	SITG7	*ITG7*	6	0	0
7	SLAM2	*LAMA2*	25	16	64
8	SLARG	*LARGE*	9	0	0
9	SPOM1	*POMT1*	27	3	11
10	SPOM2	*POMT2*	23	2	9
11	SPOMG	*POMGNT1*	18	3	17
12	SSEP1	*SEPN1*	28	2	7
Total			228	39	17
**NGS gene panel tests**					
P1	SCMDP	CMD Comprehensive	37	20	54
P2	SCO6P	Bethlem myopathy/Ullrich CMD	48	5	10
P3	SMDCP	Merosin-deficient CMD	18	17	94
P4	SMPCP	Merosin-positive CMD	2	1	50
Total			105	43	41

a Diagnostic yield expressed as a percentage of total analyzed samples; P1, Includes testing for *COL6A1, COL6A2, COL6A3, ITGA7, FKTN, FKRP, POMGNT1, POMT1, POMT2, SEPN1, LARGE, LAMA2*; P2, Includes testing for *COL6A1, COL6A2, COL6A3*; P3, Includes testing for *FKTN, FKRP, POMGNT1, POMT1, POMT2, LARGE, LAMA2*; P4, Includes testing for *COL6A1, COL6A2, COL6A3, ITGA7, SEPN1*.

## Discussion

Recent studies have highlighted the potential of NGS in mutation detection [2], [24]–[27]; however, it remains imperative to query and validate its performance efficiency prior to implementation in a clinical testing laboratory. To this end, we investigated the potential of NGS technology and its efficiency as a clinical diagnostic tool by implementing a high-throughput gene panel for CMD. Target coverage in a panel approach varies based on the target enrichment method employed prior to NGS. The relatively high sensitivity, specificity, and accuracy of target enrichment for NGS offered by the highly multiplexed PCR-based technology (RainDance) over other hybridization technologies has been previously demonstrated for CMD [23].

The evaluation of NGS performance parameters is critical to offering such diagnostic strategies in a clinical laboratory. Our study design using negative (wild-type, CMD-13) and positive controls (variant-positive samples CMD-8 to CMD-12) demonstrated the efficiency of the NGS technology in detecting all potential mutation types (Table 4). The study also established the limitations of the technology. For the entire CMD panel, there were at least 49 exons across 9 genes that had significantly low coverage (<20×) and required Sanger sequencing (Table 3). Among these 49 exons, were *POMT1* exons (19 exons) that failed amplification and hence had no coverage at all, most likely due to unusual GC content and sequence complexity. Amplification of exon 1 of most genes was problematic for similar reasons. [1], [18], [28]. Empirically, the average failure rate for target enrichment is 15–20%. Though not comparable to the low failure rates of Sanger sequencing (3%: 2 out of 63 amplicons), the NGS failure rate is compensated for by other advantages of the technology [29]. The flexibility of both batch processing and single sample processing offered by RainDance minimizes reagent wastage and maintains rapid turnaround times, even when processing is less frequently ordered for samples of rare disorders in diagnostic laboratories. One additional advantage of RainDance for target enrichment is the ability to differentiate genes from pseudogenes by the use of gene-specific primers for PCR amplification unlike other hybridization based technologies. Comparable low coverage (<10× coverage in 20% of exons) has also been observed in non-CMD genes as reported by a recent study of ataxia gene targets involving array-based enrichment and NGS sequencing [2]. Sanger sequencing may remain the strategy of choice for confirmation of low-coverage variants.

Following this successful validation, a clinical CMD NGS panel was launched at the Emory Genetics Laboratory (EGL) and has been used successfully by clinicians in CMD cases presenting with overlapping phenotypes, inconclusive biochemical studies, non-diagnostic brain or muscle MRIs. This expedited approach to molecular diagnosis avoids the diagnostic odyssey and cost associated with a serial gene testing approach. For example, on average the number of exons comprising a CMD gene is 25 (ranging from 1–65), costing around $2500 per gene for molecular analysis, clinical interpretation, and report issuance. Alternatively, the NGS-based sequencing panel offered for just $5000 includes comprehensive analysis of the current12 disease-associated genes. In addition, as more and more disease-causing genes are identified, they can be added to the panel without a significant increase in the overall cost, which is very unlikely to be the case for a gene-be-gene approach. In this study, the CMD panel approach convincingly showed better mutation detection or diagnostic yield compared to a single-gene analysis (Table 8). The efficiency and better yield of the panel approach is better illustrated by the analysis of the 20 blinded samples included in our study (Table 7). Several samples, which underwent a series of single-gene tests, and others which remained CMDs of unknown molecular etiology due to inconclusive biochemical or immunologic assays, all received a definitive diagnosis through this NGS approach. Others, in which no mutations could be detected, could either be negative for all known genes or have larger deletions or duplications requiring array CGH analysis.

Although whole-exome sequencing is making its way into clinical genetics, the high false-positive rate, long turnaround times requiring 3 to 6 months for analysis and interpretation alone, ethical challenges involving secondary findings, and the high test price when data and interpretation are included, may present prohibitive barriers to commercial application [12], [30]–[32]. Exome sequencing currently covers about 92% of the exome. From the experience of the whole exome analysis pipeline at EGL, approximately 10–20% of the 92% had low or zero coverage. The failed exons vary between individual runs and may involve genes of interest strongly associated with the patient phenotype. Given the large number of failed exons, they cannot practically be followed up and confirmed by Sanger sequencing, making the test necessarily incomplete. In contrast, targeted panel sequencing gives the laboratory the ability to complete the test by tracking all NGS failed exons and confirming by Sanger sequencing. These current limitations further highlight the significance of panel approaches over current gene-by-gene or potential whole exome-based tests. In agreement with the newly released CAP and Gargis et al. guidelines for clinical NGS validation and implementation, our study demonstrates that an NGS panel approach can be successfully adopted in clinical laboratories to improve disease diagnosis [33].

**Figure 3:**
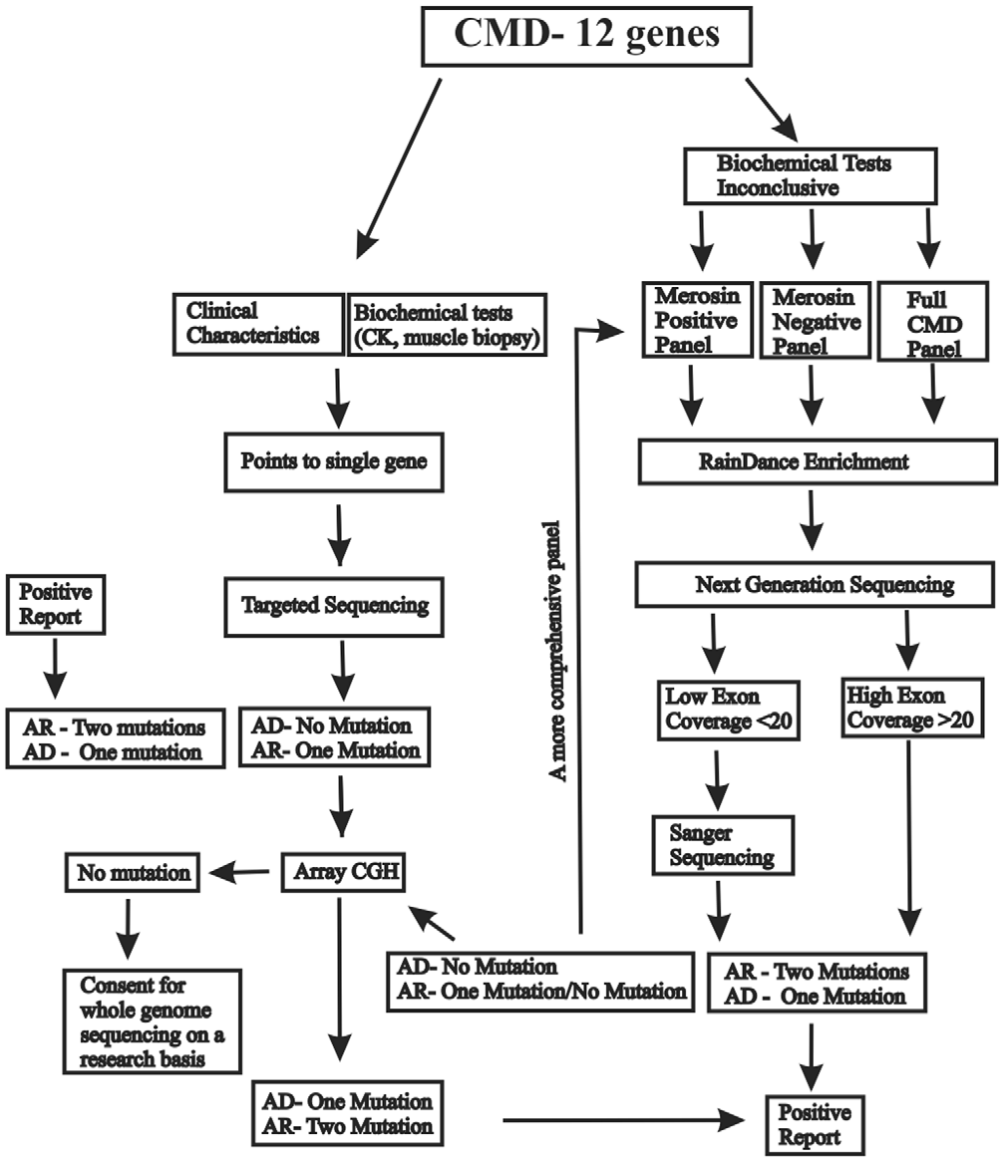
Congenital muscular dystrophy next-generation sequencing algorithm. The diagram demonstrates the diagnostic work-up of congenital muscular dystrophies using RainDance enrichment in combination with next-generation sequencing.

In conclusion, clinical CMD NGS panels offer cost-effective and more rapid turn-around molecular diagnostic testing than the conventional sequential Sanger sequencing of associated genes. A detailed step-wise approach to recommending NGS panel tests and diagnosing the disease subtype is illustrated as a flowchart (Figure 3). We anticipate that following this diagnostic algorithm will ensure a more efficient and rapid diagnosis for patients with CMD who currently lack molecular characterization.

## Materials and Methods

### Patients/Samples

To investigate whether an NGS panel can detect different types of mutations, 5 variant-positive samples (CMD-8, CMD-9, CMD-10, CMD-11, and CMD-12), each with a different type of mutation (missense, nonsense, insertion, deletion), were included in this study. These 5 variant-positive samples have been previously characterized for pathogenic variants/mutations through the currently available Sanger sequencing panels: CMD comprehensive panel in CMD-9 (all 12 CMD genes, *COL6A1*, *COL6A2*, *COL6A3*, *FKRP*, *FKTN*, *ITGA7*, *LAMA2*, *LARGE*, *POMGNT1*, *POMT1*, *POMT2*, and *SEPN1*); Bethlem myopathy/Ullrich CMD sequencing panel in CMD-8 and CMD-10 (*COL6A1*, *COL6A2*, and *COL6A3*); merosin-deficient CMD Type 1A (MDC1A) panel in CMD-11 (sequencing of *LAMA2*); and muscle-eye-brain disease (MEB) panel in CMD-12 (sequencing of *POMGNT1*). Additionally, a variant-negative wild-type control sample (CMD-13) expected to lack any pathogenic variants/mutations was also included.

To assess the clinical utility of the NGS panel, 20 samples from patients with clinically suspected CMD (diagnosed by Dr. Carsten Bonnemann, National Institutes of Health, Bethesda, MD, formerly at Children's Hospital of Philadelphia, PA) were analyzed. To this end, Emory Genetics Laboratory staff were blinded to the identity, clinical phenotype and prior genetic testing results of these 20 samples. Written informed consent was obtained from all patients and approved by the Children's Hospital of Philadelphia IRB. The clinical features of each patient are summarized in Table 1. All experiments were conducted at the Emory Genetics Laboratory (EGL), a CAP-accredited clinical laboratory.

### Primer Library Design

A primer library for target amplification of all exons of the known CMD genes was designed using the manufacturer's design parameters (RainDance Technologies) and the Primer3 algorithm (http://frodo.wi.mit.edu/primer3/). All SNPs from dbSNP build 130 were filtered from the primer selection region. The in-house primer design pipeline performed an exhaustive primer design and selection across the 65-kb targeted region. The final library consisted of primer pairs for successful amplification of 383 amplicons with Tm ranging from 57 to 59°C and primer length ranging from 16 to 21 bp.

### Target Enrichment by Droplet-Based Multiplex PCR (RainDance)

Intact genomic DNA samples were sheared to yield 3–4 kb long fragments with the Covaris S2 instrument following the manufacturer's instructions. For target sequence amplification, input DNA template mixture was prepared by mixing 1.5 µg of the above sheared DNA fragments, 4.7 µl of High-Fidelity Buffer (Invitrogen), 1.26 µl of MgSO4 (Invitrogen), 1.6 µl of 10 mM dNTP (Invitrogen), 3.6 µl of 4 M Betaine (Sigma), 3.6 µl of RDT Droplet Stabilizer (RainDance Technologies), 1.8 µl of dimethyl sulfoxide (Sigma), 0.7 µl 5 units/µl of Platinum High-Fidelity Taq (Invitrogen), and nuclease-free water to bring to a final reaction volume of 25 µl, per the RainDance protocol. These samples were then subjected to emulsification using the RDT1000 instrument (RainDance Technologies) to generate individual PCR droplets. Droplets for each sample were automatically dispensed as an emulsion into separate PCR tubes and transferred to a standard thermal cycler for PCR amplification. Samples were cycled in an Applied Biosystems GeneAmp 9700 thermocycler as follows: initial denaturation at 94°C for 2 min, 55 cycles at 94°C for 15 s, 54°C for 15 s, 68°C for 30 s, final extension at 68°C for 10 min, and a 4°C hold.

After PCR amplification, an equal volume of RDT 1000 Droplet Destabilizer (RainDance Technologies) was added to each emulsion PCR tube, vortexed for 15 s, and centrifuged at 13,000 g for 5 min. The oil from below the aqueous phase was carefully removed from the sample. The remaining sample was then purified using a MinElute column (Qiagen) following the manufacturer's recommended protocol.

### NGS Sequencing by SOLiD3

The ends of the amplicons were blunt end-repaired by adding the reagents to the purified DNA (diluted to 68 µl): 10 µl 10× Blunting Buffer (Epicentre), 10 µl 2.5 mM dNTP Mix (Invitrogen), 10 µl 10 mM ATP, 2 µl End-it enzyme mix (Epicentre), and sterile water to a total reaction volume of 100 µl. The reaction was incubated at room temperature for 30 min, and the DNA was immediately purified using Ampure XP beads (Agencourt). The amplicons were then concatenated using the NEB Quick Ligation kit according to the manufacturer's protocol. DNA was purified using Ampure XP beads and eluted in 105 µl of low TE. An Agilent 7500 Bioanalyzer chip was run to confirm the concatenation of PCR products. The sample was then fragmented and processed as described in the manufacturer's standard SOLiD3 workflow (Applied Biosystems).

### Validation of Variants and Mutations by Sanger Sequencing

Exon-specific primers were designed to amplify individual exons along with 20–50 bp of flanking intronic regions on either side for *LAMA2*, *COL6A1*, *COL6A2*, *COL6A3*, *FKTN*, *POMGnT1*, *POMT1*, *POMT2*, *FKRP*, *LARGE*, *ITGA7*, and *SEPN1*. Samples were prepared by fluorescence sequencing on the ABI 3730XL DNA analyzer with BigDye Terminator chemistry and the BigDye XTerminator purification kit (Applied Biosystems). Individual sequences were aligned against reference sequences (downloaded from NCBI) using Mutation Surveyor v3.30 software (SoftGenetics) and analyzed for variations or mutations.
